# Genome-wide survey reveals the genetic background of Xinjiang Brown cattle in China

**DOI:** 10.3389/fgene.2023.1348329

**Published:** 2024-01-12

**Authors:** Xiao Wang, Zhen Ma, Liang Gao, Lixin Yuan, Zhibing Ye, Fanrong Cui, Xiaoping Guo, Wujun Liu, Xiangmin Yan

**Affiliations:** ^1^ College of Animal Science, Xinjiang Agricultural University, Urumqi, China; ^2^ Yili Vocational and Technical College, Yili, China; ^3^ Institute of Animal Science, Xinjiang Academy of Animal Science, Urumqi, China; ^4^ Yili Kazakh Autonomous Prefecture General Animal Husbandry Station, Yili, China

**Keywords:** Xinjiang Brown cattle, specific-locus amplified fragment-sequencing, genetic structure, genetic diversity, candidate genes, ancestry proportion

## Abstract

**Introduction:** Xinjiang Brown cattle are a famous dual-purpose (dairy-beef) cultivated breed in China that occupy a pivotal position within the cattle breeding industry in Xinjiang, China. However, little information is available on the genetic background of this breed. To fill this research gap, we conducted a whole-genome screen using specific-locus amplified fragment sequencing to examine the genetic structure and diversity of 130 Xinjiang Brown cattle-grazing type (XBG, traditional type) cattle.

**Methods:** A subsequent joint analysis incorporating two ancestral breeds, specifically 19 Brown Swiss (BS) foreign and nine Kazakh (KZ) Chinese cattle, as well as 20 Xinjiang Brown cattle-housing type (XBH) cattle, was used to explore the genetic background of the Xinjiang Brown cattle.

**Results:** The results showed that, after nearly a century of crossbreeding, XBG cattle formed a single population with a stable genetic performance. The genetic structure, genetic diversity, and selection signature analysis of the two ancestral types showed highly different results compared to that of XBH cattle. Local ancestry inference showed that the average proportions of XGB cattle within the BS and KZ cattle lineages were 37.22% and 62.78%, respectively, whereas the average proportions of XBH cattle within the BS and KZ cattle lineages were 95.14% and 4.86%, respectively. Thus, XGB cattle are more representative of all Xinjiang Brown cattle, in line with their breeding history, which involves crossbreeding. Two complementary approaches, fixation index and mean nucleotide diversity, were used to detect selection signals in the four aforementioned cattle breeds. Finally, the analysis of 26 candidate genes in Xinjiang Brown cattle revealed significant enrichment in 19 Gene Ontology terms, and seven candidate genes were enriched in three pathways related to disease resistance (*CDH4*, *SIRPB1*, and *SIRPα*) and the endocrine system (*ADCY5*, *ABCC8*, *KCNJ11*, and *KCNMA1*). Finally, development of the core SNPs in XBG cattle yielded 8,379 loci.

**Conclusion:** The results of this study detail the evolutionary process of crossbreeding in Xinjiang Brown cattle and provide guidance for selecting and breeding new strains of this species.

## 1 Introduction

**FIGURE 1:**
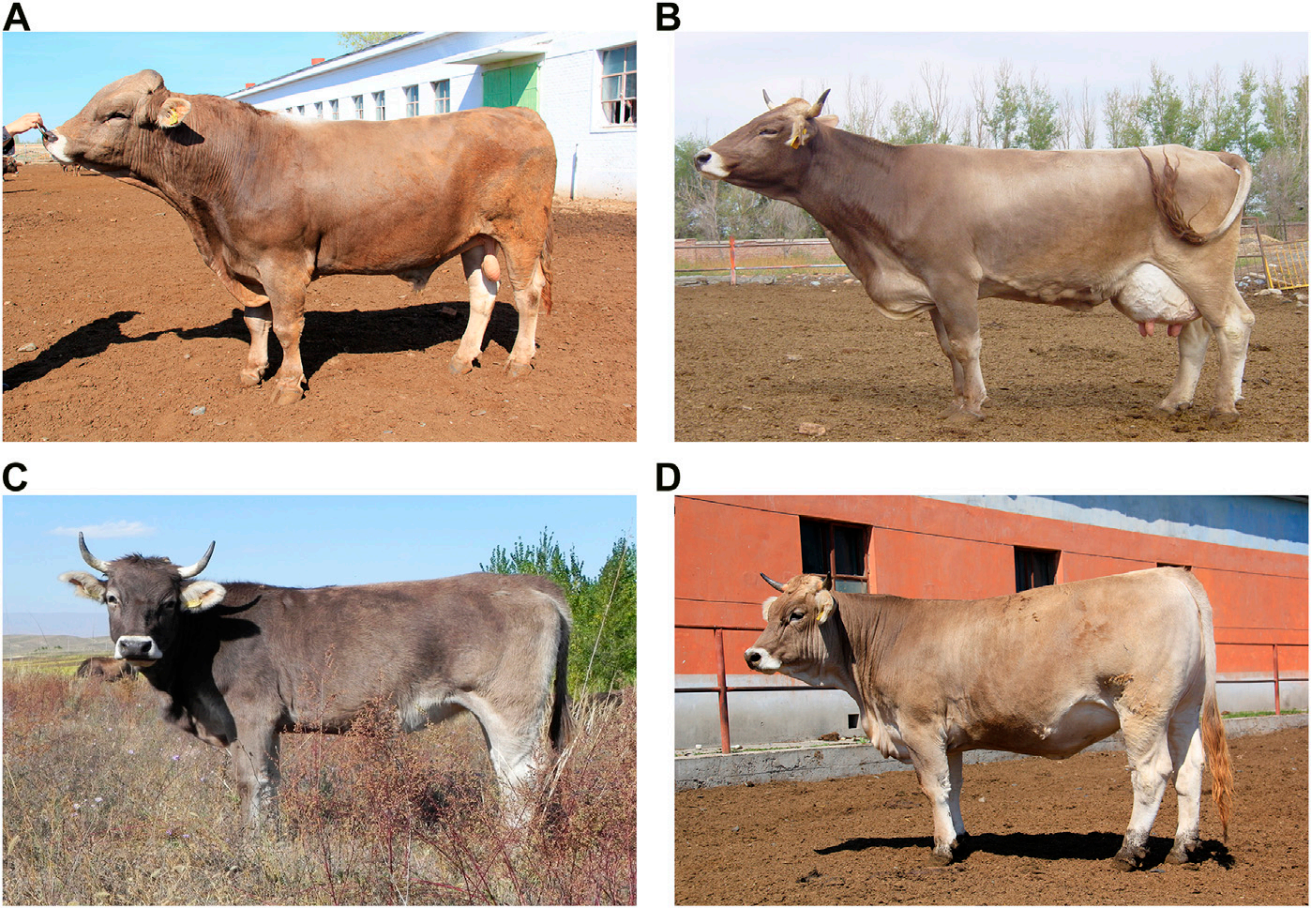
Xinjiang Brown cattle photos. **(A)** Xinjiang Brown cattle breeding bull in Yili-Tacheng region. **(B)** Xinjiang Brown cattle breeding cow in Yili-Nilka County. **(C)** Xinjiang Brown cattle-grazing type (XBG) cow in Yili-Tacheng region. **(D)** Xinjiang Brown cattle-housing type (XBH) cow in the Urumqi breeding farm.

Xinjiang Brown cattle is a dual-purpose (dairy-beef) cultivated breed bred independently in China. These cattle exhibit strong adaptability, superior grazing, and production performance within the extreme arid, cold, and barren environment of northern Xinjiang (Zhou et al., 2019) (Figures 1A, B). These qualities make them an important cattle breed for local breeders, farmers, and herders. By 2022, the stock of purebred Xinjiang Brown cattle amounted to approximately 1,169 thousand heads. Specifically, 27 thousand heads were in feedlots, whereas 1,142 thousand of them were in pastures. In addition, there were specifically 760 thousand and 14 thousand fertile cows and bulls, respectively. Overall, this stock accounted for approximately 1/5th of all types of cattle stock in Xinjiang. Xinjiang Brown cattle were selectively bred during the early 20th century, in which the Kazakh (KZ) cow was the female parent and underwent three-stage hybridization with the Brown Swiss (BS) bull or the Kostroma or Ala-Tau bulls, two breeds of Brown Swiss cattle origin (Yurchenko et al., 2017). As a result, the Ministry of Agriculture of China certified the Xinjiang Brown as a novel dual-purpose (dairy-beef) cattle breed in 1983 (Zhang et al., 2022). In the following 40 years, this breed has also transitioned into the expansion phase of selection and improvement (1987–2006), followed by a breeding phase of specialized strains (2007 to present). With the introduction of frozen semen from BS bulls primarily from Germany, the United States of America, and Canada, Xinjiang Brown cattle have been further improved and various new strains have been bred to meet the demands of the market and socio-economic development. Nearly 97% of Xinjiang Brown cattle are reared under semi-herding and grazing conditions and are referred to in this study as the Xinjiang Brown cattle-grazing type (XBG) cattle (Figure 1C). The XBG population is large and has been less affected by frozen sperm from BS cattle since 2007, which has helped the genetic preservation of the original makeup of Xinjiang Brown cattle. However, the genetic background of XBG cattle characteristics remains mostly unknown at the genomic level and the extent to which their breed contribution is influenced by Chinese KZ or foreign BS breeds is uncertain.

In recent years, developments in genomic technology have facilitated genomic analyses that have enabled access to individual DNA information via whole-genome sequencing (WGS) (Yin et al., 2019) and genotype-by-sequencing (GBS) (Elshire et al., 2011). Research on Xinjiang Brown cattle has also entered the omics era, as evidenced by genome-wide association studies for milk production and reproductive traits (Zhou et al., 2019), genome-wide identification and analysis of long non-coding RNAs in the longest dorsal muscle tissue (Yan et al., 2021), and genomic selection for milk production traits (Zhang et al., 2022). A WGS analysis was also performed to study the genetic evolution of Xinjiang Brown cattle, which more comprehensively revealed their genetic background, genetic diversity, and adaptive mechanisms (Chen et al., 2022a); however, the samples used in this study were not genuinely representative, as all 50 samples were collected from Xinjiang Brown cattle-housing type (XBH) cattle (Figure 1D) at the Urumqi breeding farm, XBH accounts for less than 3 percent of the total number of Xinjiang Brown cattle. Additionally, the XBH contains a disproportionately high percentage of BS genetic lineage and has low genetic diversity and closely related individuals as confirmed by trial results. Hence, gathering an extensive and more inclusive group of XBG cattle to obtain fresh test outcomes from is imperative. Additionally, the findings of the previous samples from XBH cattle should be blended with new results to reflect the genetic background of Xinjiang Brown cattle more precisely and impartially.

To this end, in this study, we used specific-locus amplified fragment (SLAF) sequencing (SLAF-seq) (Zhou and Pan, 2023) techniques to obtain individual DNA information from 130 XBG cattle. The published data for KZ, BS, and XBH cattle from 48 WGS analyses were downloaded from NCBI and combined with the SLAF-seq data of 129 XBG cattle for joint analysis; the proven feasibility of merging two types of data has previously confirmed the findings of an exploration of olive diversity in plants (Friel et al., 2021) and an assessment of the adaptation of Nigerian cattle in animals (Mauki et al., 2022). This study focused on analyzing the genetic structure, genetic diversity, and selection signatures of the XBG population and aimed to establish a molecular basis that could assist in the conservation, scientific introduction, and selection of breeding resources for Xinjiang Brown cattle.

## 2 Materials and methods

### 2.1 Sample collection

The study team visited the primary production area of XBG in the Yili Tacheng region in 2021. We collected blood samples from 130 XBG cattle on a large private ranch, despite difficulties in sampling resulting from grazing conditions. Whole-blood samples (10 mL) from 130 XBG cattle (all females) were divided into four groups depending on the color of the cow’s coat (group A = 40, B = 76, C = 10, and D = 4). The coat color of group A was a normal brown, that of B was dark brown, with fawn for C, and light brown for D. Genomic DNA extractions were performed using the phenol–chloroform method (Sambrook and Russell, 2006) at the Xinjiang Academy of Animal Science. The DNA purity of the extracted samples was determined via quantification using a Thermo Scientific™ NanoDrop 2000 spectrophotometer (Thermo Scientific, United States). Furthermore, the quality of the DNA extracts was assessed by subjecting them to electrophoresis on a 2% agarose gel against a 2 kilobase (kb) DNA ladder marker. The 130 samples were then subjected to sequencing on the SLAF platform.

### 2.2 SLAF library construction and sequencing

The SLAF library was created as described previously with minor adjustments (Aerts et al., 2013). To ensure the anticipated SLAF output, we avoided repetitive SLAFs and selected a relatively uniform distribution of restriction fragments in the genome. Next, we conducted a simulated restriction enzyme digestion on the existing *B. taurus* genome (UMD 3.1) (Zimin et al., 2009). The genomic DNA from each sample was digested using a combination of RsaI and HaeIII restriction enzymes. This was followed by adding a single nucleotide (A) overhang to the 3′ end of the SLAF tags. To ensure ligation of dual-index sequencing adapters to A-tailed tags, we carried out restriction-ligation reactions using T4 DNA ligase (New England Biolabs). Subsequently, DNA amplification was performed using PCR and the resulting products were purified using the E. Z.N.A.H Cycle Pure Kit (Omega). The purified samples were combined and incubated with two specified restriction enzymes, RsaI and HaeIII. After being ligated with ATP and a Solexa adapter at the paired-end, the reaction was purified using a Quick Spin column (Qiagen, Venlo, Netherlands) and segregated on a 2% agarose gel. Fragments between 450 and 480 bp were extracted using a Gel Extraction Kit (Tiangen, China). These SLAFs were then subjected to PCR for barcode addition. The amplified DNA samples underwent re-purification before being prepared for 150-base paired-end sequencing using an Illumina NovaSeq6000 sequencing platform (Illumina, San Diego, CA, United States) at Biomarker Technologies Corporation (Beijing, China).

### 2.3 WGS library construction and sequencing

Forty-eight publicly available WGS genome datasets were acquired from previous studies (Chen et al., 2018; Chen et al., 2022a). Raw sequencing data of XBH (*n* = 20), KZ (*n* = 9), and BS (*n* = 19) cattle are available at NCBI BioProject ID: PRJNA833533, PRJNA379859, and PRJEB28191, respectively (Supplementary Table S1).

### 2.4 Processing, mapping, filtering, and single-nucleotide polymorphism calling of SLAF reads

All indexed sequenced reads with clear information were clustered based on sequence similarity. Similarity clustering was used to group the sequenced reads from the same locus (Jones et al., 2013) and aligned to a reference genome (UMD 3.1) (Zimin et al., 2009) using BWA v0.7.17 software (Li and Durbin, 2009a). Single-nucleotide polymorphism (SNP) calling was performed using GATK v3.8 software (McKenna et al., 2010) and SAMtools v1.3.1 software (Li et al., 2009b). In addition, the filtered high-quality SNPs were used to annotate the SNP detection results using SnpEff software (Cingolani et al., 2012), which can provide the region of the genome where the variant locus occurs (intergenic region, gene region, or CDS region, etc.) and the effect of the variant (synonymous non-synonymous mutation, etc.).

### 2.5 Population genetic structure and genetic diversity analysis of SLAF datasets

The high-confidence SNPs produced via the above procedures were used to infer the genetic structure of the 130 XBG cattle. We constructed an unrooted phylogenetic tree using the neighbor-joining method with the Kimura 2-parameter/p-distance model in MEGA-CC (MEGAX) software (Kumar et al., 2018), with 1,000 bootstrap replicates. Principal components analysis (PCA) was performed using the smartPCA module of EIGENSOFT v7.2.0 software, using the default parameters (Price et al., 2006). Estimation of the genetic relationships from SNPs using one of the five main functions of GCTA v1.91.7 software (Yang et al., 2011) to estimate the kinship between two individuals of a natural population is possible. The population structure within the 130 XBG cattle was inferred using ADMIXTURE v1.3.0 software (Alexander et al., 2009), with K values (the putative number of populations) ranging from 1 to 10. The optimal number of clusters K (best taxa) was determined as the one with the minimum cross-validation error rate. The Q matrix for each K value within stacked assignment bar plots was generated using the R package “Pophelpers” (Francis, 2017). Pi values were calculated using VCFtools v0.1.16 software based on the high-confidence filtered SNPs and a 100 kb window with a step size of 10 kb for each sub-population (Danecek et al., 2011).

### 2.6 Processing, mapping, filtering, SNP calling, and data merging of SLAF and published WGS reads

Raw paired-end reads of the 129 XBG SLAF-seq genome datasets and 48 publicly available WGS genome datasets were mapped to the *B. taurus* reference genome (ARS-UCD 1.2) (Rosen et al., 2020) using BWA v0.7.17 software (parameters: mem -t 4 -k 32 -M) (Li and Durbin, 2009a). SNP calling was performed using both GATK v3.8 software (McKenna et al., 2010) and SAMtools v1.3.1 software (Li et al., 2009b) (WGS parameter: rmdup; SLAF parameter: sort) analyses, and a locus was defined as a SNP if it was simultaneously called from these two packages. The “mpileup” command was used to identify SNPs with the parameters “-q 1 -C 50 -S -D -m 2 -F 0.002 -u”. Then, to exclude SNP calling errors caused by incorrect mapping, only high-quality SNPs [coverage depth ≥ 4, RMS mapping quality ≥ 20, minor allele frequency (MAF) ≥ 0.01, miss ≤ 0.3] were retained for subsequent analysis. BCFtools v1.7 software was used to merge overlapping genomic regions between the SLAF and WGS datasets (Li et al., 2009b).

### 2.7 Population genetic structure and genetic diversity analysis of the merged SLAF and published WGS datasets

An individual-based neighbor-joining tree was constructed for the 177 evaluated cattle based on the p-distance, with one outgroup (i.e., *Bos mutus*) (Supplementary Table S1), using TreeBest v1.9.2 software (Vilella et al., 2009) with 1,000 bootstrap replicates. We further conducted PCA to evaluate genetic structures using GCTA v1.91.7 software (Yang et al., 2011). The population genetic structure was examined using ADMIXTURE v1.3.0 software (Alexander et al., 2009), and the number of assumed genetic clusters K ranged from 2 to 8, with 10,000 iterations for each run. In addition, RFMix v2.03 software (Maples et al., 2013) was used for Local-Ancestry inferencing. The indicators of observed heterozygosity (Ho), expected heterozygosity (He), polymorphism information content (Pic), and Nei’s genetic diversity index (Nei) analysis were counted using the Stacks v1.45 populations program (Catchen et al., 2013).

### 2.8 Genome-wide selective sweep test, Gene Ontology annotation, and Kyoto Encyclopedia of genes and genomes functional enrichment

We used VCFtools v0.1.16 software (Danecek et al., 2011) to calculate Pi and the fixation index (FST) with the window size set to 50 k and sliding window 10 k. The genome-wide distribution of FST values and mean Pi (θπ) ratios for the indicated group pairs were computed to discover genome-wide selection sweeps linked to cattle adaptability. The FST values were Z-transformed as Z (FST) = (FST—µFST)/σFST, where σFST represents the FST standard deviation and µFST is the FST mean. Log2-transformed θπ ratios were obtained. The empirical percentiles of Z (FST) and log2 (θπ ratio) in each window were then computed and ranked. Under strong selective sweeps, we looked at the windows that simultaneously had the top 5% Z (FST) and log2 (π ratio) values as potential outliers. Every outlier window had a corresponding SNP and gene assigned to it (the selection method of the candidate window was modified according to the actual situation).

Protein-coding genes were functionally annotated through the utilization of BLASTp (with an E-value of <10–5) (Gish and States, 1993), with protein sequence databases sourced from SwissProt. Gene Ontology (GO) (Ashburner et al., 2000) enrichment analysis of differentially expressed genes was implemented via the GOseq R package (Young et al., 2010), in which gene length bias was corrected. GO terms with a corrected *p*-value less than 0.05 were considered significantly enriched by differentially expressed genes. We used KOBAS (Mao et al., 2005) to test the statistical enrichment of differentially expressed genes in Kyoto Encyclopedia of Genes and Genomes (KEGG) pathways. Pathways with a q-value < 0.05 were considered significantly enriched.

### 2.9 Core SNP development

The SNP markers of the 129 XBG cattle were screened for core markers. The first step of this screening process was depth filtering, during which SNP loci with a depth of at least ×4 were retained in this project and the low depth loci were filtered out. The second step was completeness filtering for markers with poor genotypic integrity coverage; markers with a genotypic coverage of at least 70% of all individuals in the population were retained (Tian et al., 2015; Gao et al., 2016). The third step was MAF filtering, which filtered out loci with MAF values below 0.01. The fourth step was to filter by Pic value; the Pic value was calculated as Pic = 1–∑fi^r^, where fi is the gene frequency of locus I. Loci with Pic values less than 0.4 were filtered out. Finally, loci located in the intergenic region based on the functional annotation of the SNP loci were filtered out, retaining only loci located in the upstream and downstream areas of the gene and within the gene SNP loci (Zhang et al., 2013; Graebner et al., 2015).

## 3 Results

### 3.1 SLAF tag development

**FIGURE 2:**
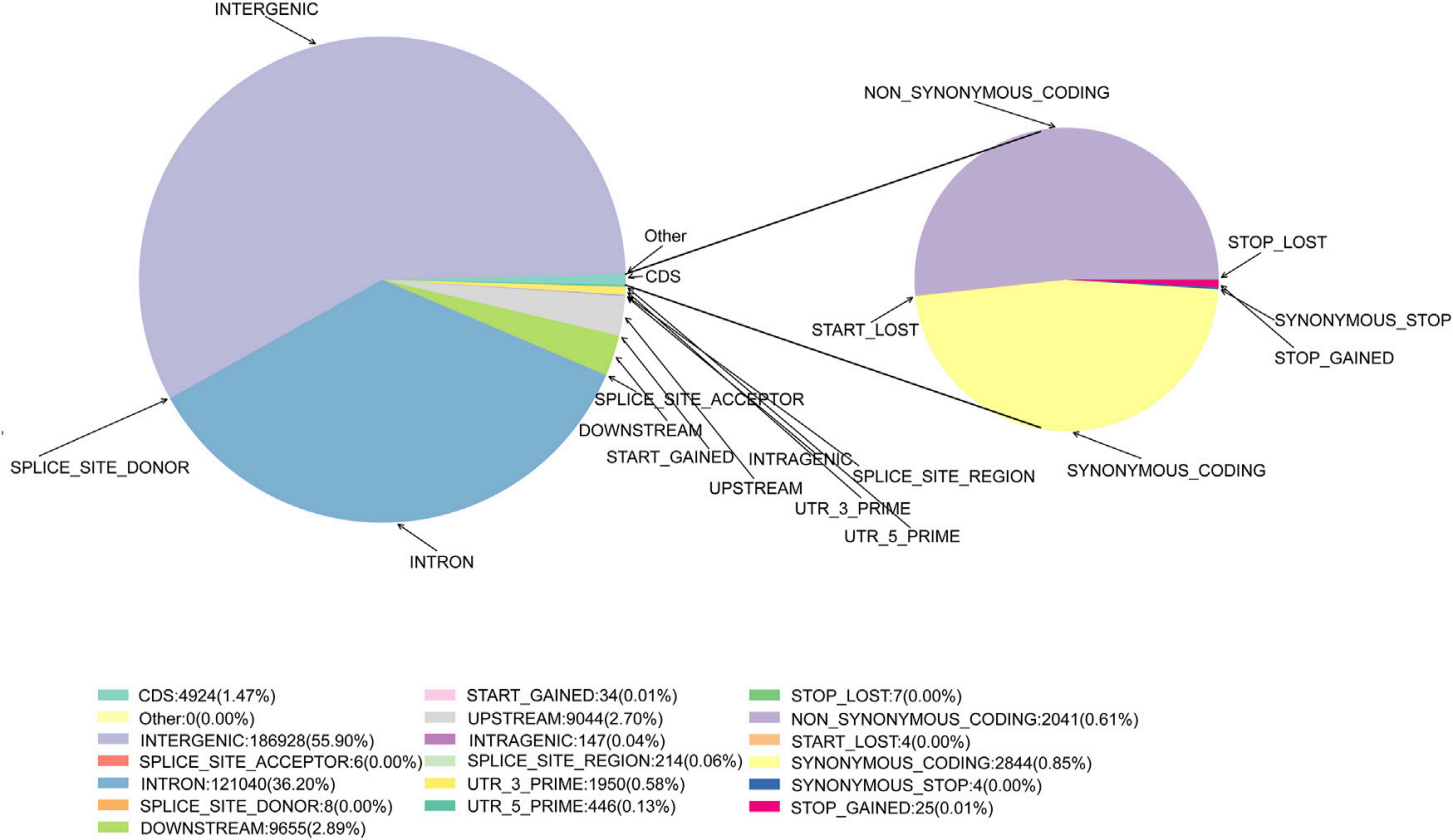
The position of the SNPs in gene structures and annotations of the SNPs in the exons.

Following quality control and data filtering, a total of 130 libraries were constructed for the cattle genomic DNA samples. These libraries yielded 657.79 Mb clean reads from SLAF-seq. The total number of reads obtained from each sample ranged from 1,661,853 to 10,777,757. The GC content was 42.70%–49.16%, with an average of 46.02%, and the 3 M quality score of the sequenced bases was 87.97%–94.21%, with an average of 92.85% (Supplementary Table S2). A total of 984,712 SLAF tags were designed, with an average sequencing depth of 12.36X (Supplementary Figure S2; Supplementary Table S3). There were 28,208 polymorphic SLAF tags with a total of 4,839,549 SNP markers. SNP integrity was 22.52%–45.90%, and heterozygosity was 4.86%–11.04% (Supplementary Figures S2, S3; Supplementary Tables S4, S5). Further analysis of SNP distribution in the genome revealed that 55.90% were in intergenic regions, with 0.04% in intragenic regions, 36.20% in introns, and 2.70% and 2.89% in the 5 kb regions upstream and downstream, respectively (Figure 2, Supplementary Table S6). These potential functional SNPs provide valuable genetic resources for exploring the genetic background of the Xinjiang Brown cattle.

### 3.2 Population genetic structure and genetic diversity of XBG

**FIGURE 3:**
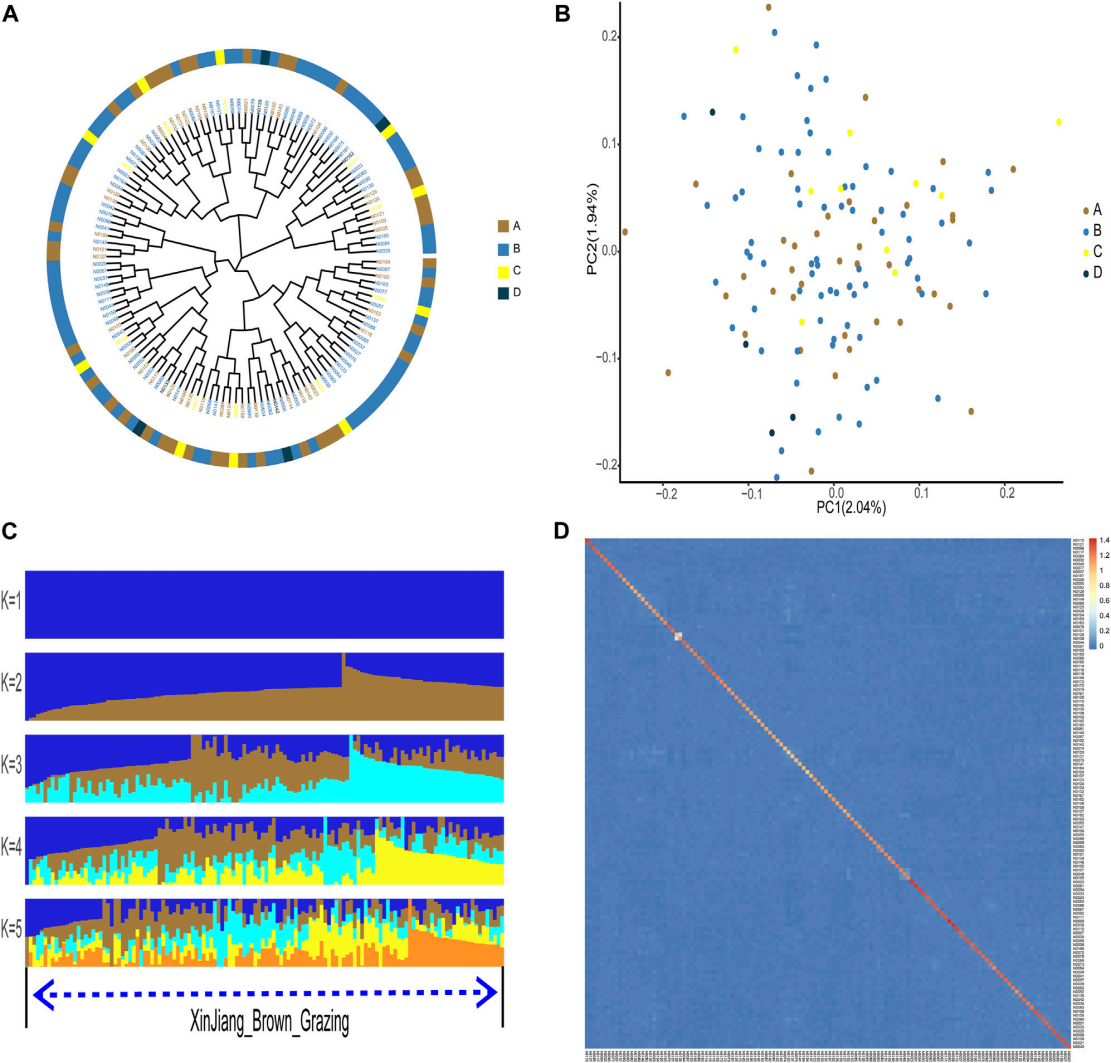
Phylogenetic relationship, population structure, and individual kinship of the Xinjiang Brown cattle-grazing type (XBG) cattle among the four cow coat color groups in this study. **(A)** Neighbor-joining phylogenetic tree constructed from single-nucleotide variant data among the four color groups. **(B)** Principal component analysis for the first two PCs of the 130 XBG cattle. **(C)** ADMIXTURE analysis with five presumed ancestral groups to one presumed ancestral group (K = from 1 to 5). **(D)** Heat map of kinship values for the 130 individual XBG cattle.

According to phylogenetic analyses, the four-colored groups of XBG exhibit heterogeneity (i.e., no apparent clustering) (Figure 3A). Hree-dimensional PCA showed that the first, second, and third axis captured 1.19%, 1.14%, and 1.03% of the overall variance, respectively, and that the four-colored groups of XBG cattle are mostly clustered, a trend that is congruent with the PCA results (Figure 3B). The genetic structures of the four-colored groups of XBG were analyzed across different clusters (K from 1 to 10) using the cross-validation error rate (Figure 3C; Supplementary Figure S4). The cross-validation error rate was lowest when K = 1 (Supplementary Figure S5), confirming the lack of genetic differentiation among the four-colored groups; this finding is congruent with that obtained via the PCA and phylogenetic analyses. The heat map of kinship values is uniformly blue, indicative of the 130 XBG individuals being distantly related to each other (Figure 3D).

The observed heterozygosity (Ho), expected heterozygosity (He), polymorphism information content (Pic), and minor allele frequency (MAF) were calculated for the four XBG color groups based on SNP loci used to characterize the genetic diversity of the different cattle populations (Pan et al., 2016). The average Ho, He, Pic, and MAF of the four XBG groups were 0.2364, 0.2718, 0.2216, and 0.2241, respectively (Table 1).

**TABLE 1:** Genetic diversity of the grazing type of Xinjiang Brown cattle among the four cow coat color groups.

Group	Ho	He	Pic	MAF
A	0.2371	0.2872	0.2355	0.2047
B	0.2270	0.2878	0.2364	0.2040
C	0.2297	0.2691	0.2191	0.2228
D	0.2519	0.2431	0.1956	0.2651
Average	0.2364	0.2718	0.2216	0.2241

Abbreviations: He, expected heterozygosity; Ho, observed heterozygosity; MAF, minor allele frequency; Pic, polymorphism information content.

### 3.3 Merged SLAF and published WGS datasets for population genetics analyses

The 48 publicly available genomes were downloaded from the NCBI database. Following quality control, the resultant high-quality (Q20 ≥ 90%, Q30 ≥ 85%) data (clean data) amounted to 165,561 gigabytes (Gb). Furthermore, the GC distribution of the resultant sequence data was normal, and none of the 48 samples were contaminated, allowing for their use in subsequent analyses (Supplementary Table S7). The reads were then aligned to the taurine reference genome (*B. taurus* ARS-UCD 1.2) (Rosen et al., 2020) and merged with 129 SLAF genomes for genotyping. A total of 22,708,388 raw SNPs were detected in 177 samples; the data were then filtered using Dp4-miss0.3-maf0.01 for the WGS and SLAF samples respectively, resulting in 17,201,439 SNPs from the former and 167,936 from the latter. The two SNP datasets were then merged to yield 104,163 common loci, which were once again filtered using the Dp4-miss0.3-maf0.01 condition. Ultimately, 99,933 high-quality SNP loci were obtained for subsequent analysis.

### 3.4 Population genetic structures of Xinjiang Brown cattle and their ancestor species

**FIGURE 4:**
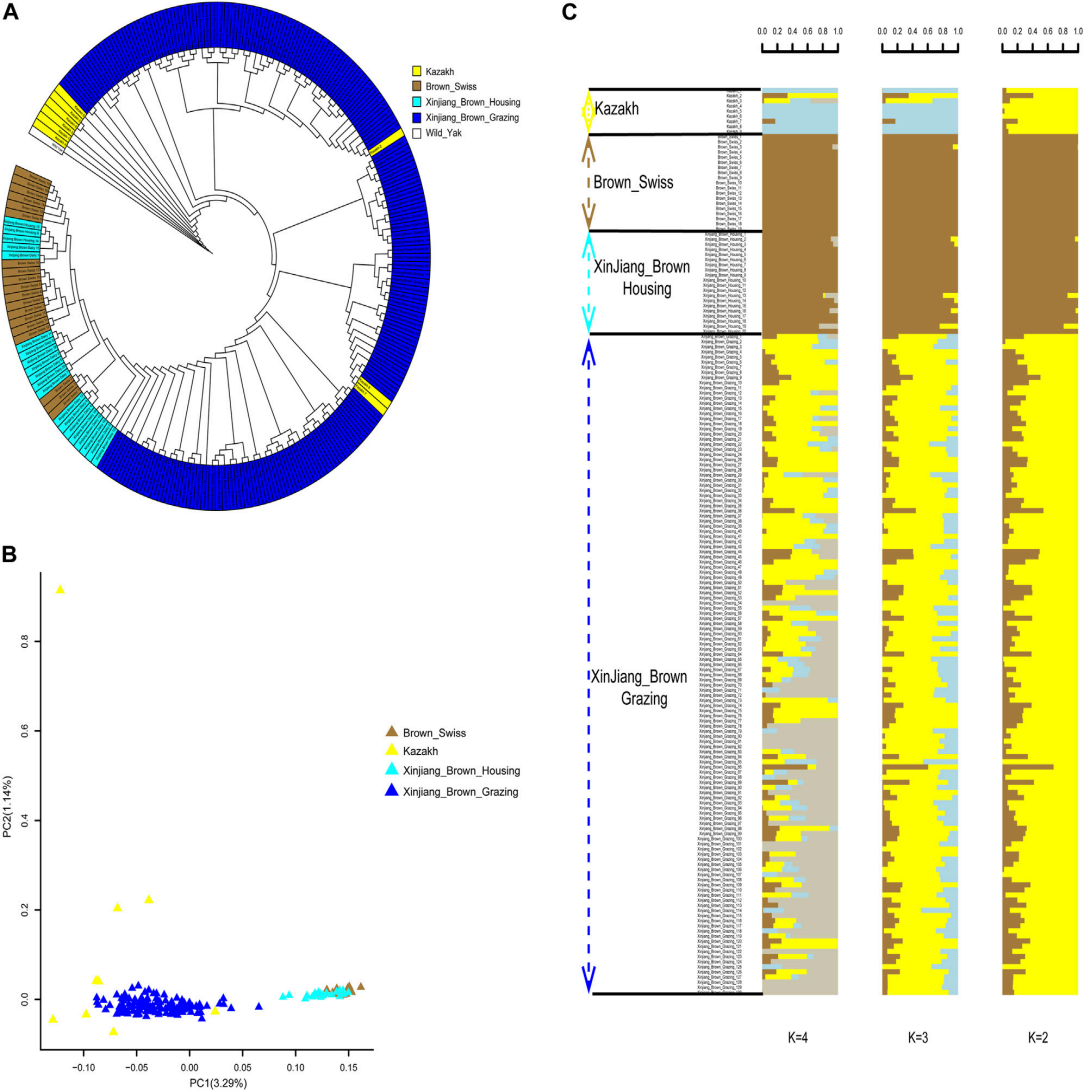
Phylogenetic relationship and population structure of the Xinjiang Brown cattle-grazing type (XBG) cattle and the other three breeds evaluated in this study. **(A)** Neighbor-joining phylogenetic tree constructed from single-nucleotide variant data among four populations. **(B)** Principal component analysis for the first two PCs of the 177 studied cattle. **(C)** ADMIXTURE analysis with four presumed ancestral groups to two presumed ancestral groups (K = from 2 to 4).

Phylogenetic trees yielded clear genetic structure, with XBG shown to be most closely related to KZ. In contrast, XBH and BS were found to be genetically indistinguishable. The closest relatives of the XBG were a mixture of KZ and BS cattle (Figure 4A). PCA also mainly distinguished the two clusters along PC1 (i.e., with two clusters being brought forth: XBG & KZ and XBH & BS). KZ were further separated from XBG populations along PC2, but the separation was incomplete (Figure 4B). Furthermore, ADMIXTURE analysis assuming ancestral number K from 2 to 8 was performed (Figure 4C; Supplementary Figure S6), and we found that the cross-validation error rate was lowest when K = 2 (Supplementary Figure S7), allowing for an inference of the genetic structure and admixture specifically for the two cattle clusters.

### 3.5 Global and local ancestry proportion

We determined the global ancestry proportions of the cattle using ADMIXTURE analysis for XBG, XBH, and their parents based on the breeding procedure. The findings revealed that when K = 2, ancestor 1 and ancestor 2 were used, the average proportions in XBG were 19.05% and 80.95%, in XBH they were 97.96% and 2.04%, in BS they were 99.97% and 0.03%, and in KZ they were 10.21% and 89.78% (Figure 4C).

**FIGURE 5:**
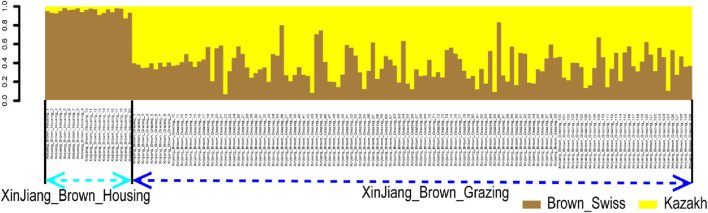
Ancestry proportion of the 129 XBG and 20 XBH individuals inferred using RFMix, as based on the reference panels of Kazakh and Brown Swiss cattle.

Additionally, a rapid and reliable forward-backward technique in RFMix was used to perform local ancestry inference in the context of XBG and XBH. Thus, in the context of BS and KZ lineages, the average proportions of XBG were 37.22% and 62.78%, respectively. In addition, these two bloodlines had average XBH proportions of 95.14% and 4.86%, respectively (Figure 5).

### 3.6 Population genetic diversity of Xinjiang Brown cattle and their ancestor species

The KZ cattle had the highest Ho, He, Nei’s genetic diversity index (Nei), and Pic values, indicating that this population had the highest genetic diversity. The XBH population had the lowest Ho, He, Nei, and Pic values, indicating that this population had the lowest genetic diversity (Table 2).

**TABLE 2:** Genetic diversity in the four studied cattle breeds.

Population (breed)	Ho	He	Nei	Pic
Kazakh	0.2496	0.2368	0.2368	0.2512
Xinjiang_Brown_Grazing	0.2066	0.2204	0.2204	0.2214
Brown_Swiss	0.1919	0.1810	0.1810	0.1861
Xinjiang_Brown_Housing	0.1812	0.1745	0.1754	0.1831

Abbreviations: He, expected heterozygosity; Ho, observed heterozygosity; Nei, Nei’s diversity index; Pic, polymorphism information content.

### 3.7 Identification and functional annotation of candidate genes using a selective sweep test

**FIGURE 6:**
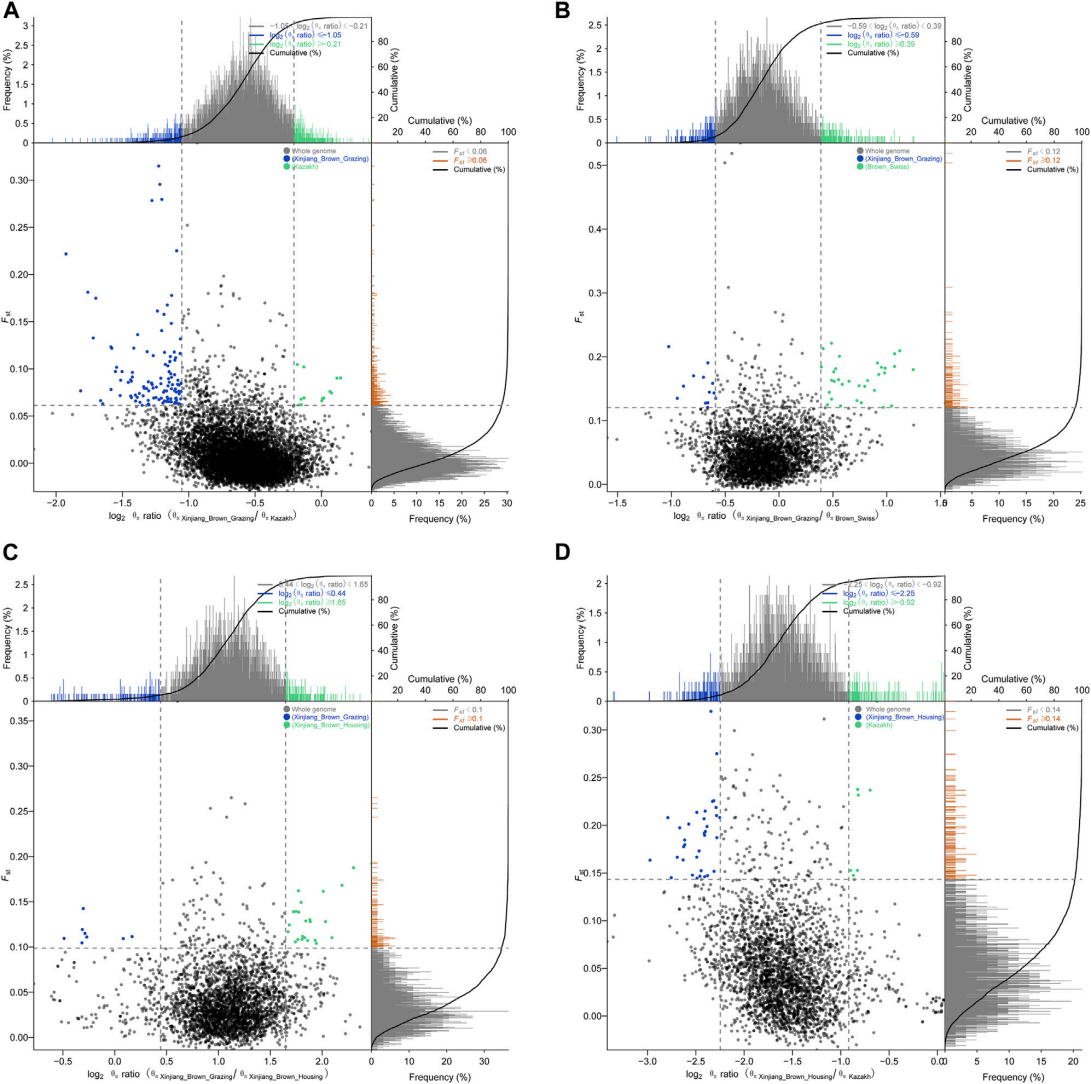
Schematic diagrams of selection signals. **(A)** Xinjiang Brown cattle-grazing type vs. Kazakh cattle. **(B)** Xinjiang Brown cattle-grazing type vs. Brown Swiss cattle. **(C)** Xinjiang Brown cattle-grazing type vs. Xinjiang Brown cattle-housing type. **(D)** Xinjiang Brown cattle-housing type vs. Kazakh cattle.

**FIGURE 7:**
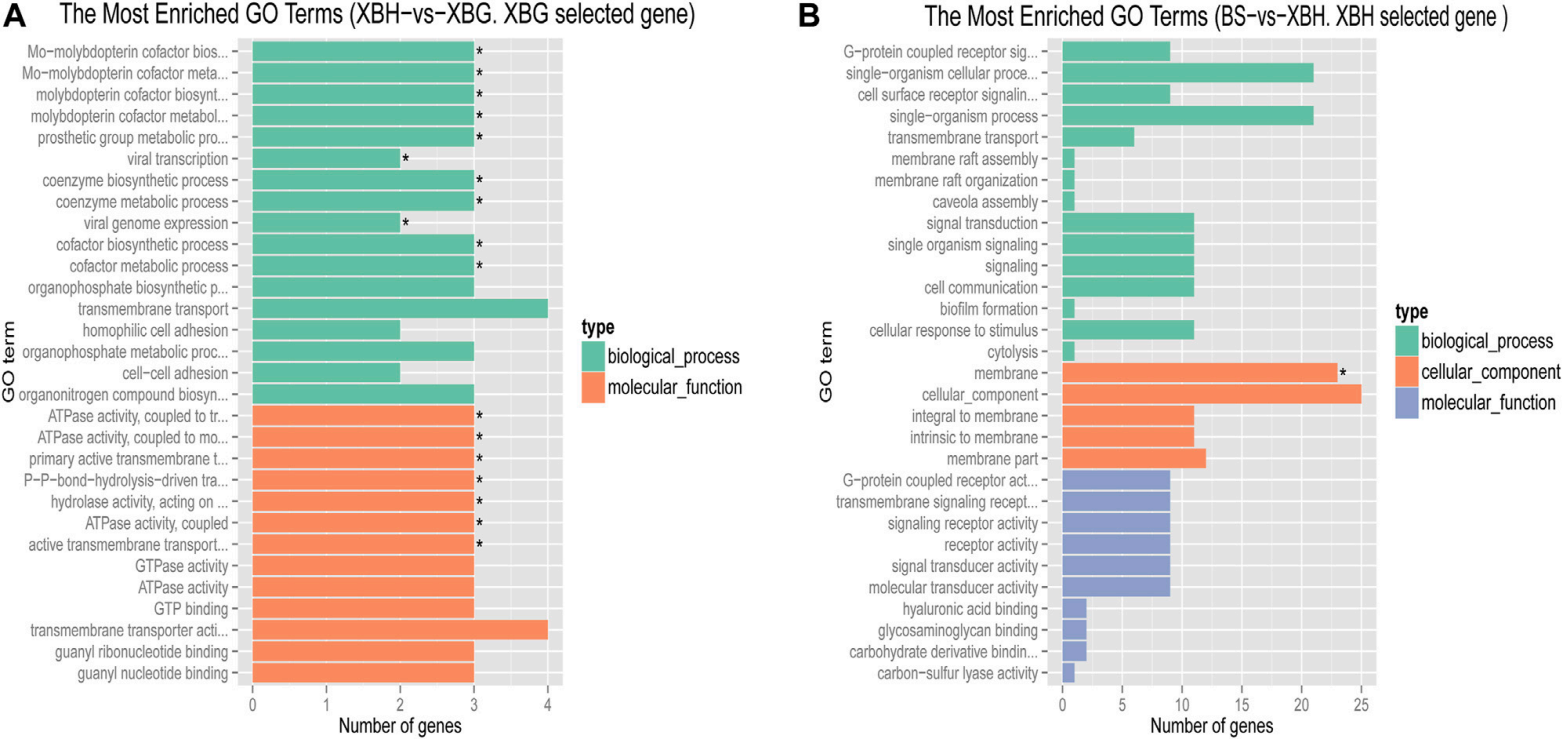
Bar chart of the distribution of candidate genes in different GO categories. **(A)** The most enriched GO terms of a Xinjiang Brown cattle-grazing type (XBG) selected gene in Xinjiang Brown cattle-housing type (XGH) vs. XBG. **(B)** The most enriched GO terms of an XBH selected gene in Brown Swiss (BS) cattle vs. XBG.

Pairwise comparisons of the four cattle breeds were calculated using selective sweep approaches. The calculated FST (Supplementary Figure S9) and θπ (Supplementary Figure S8) values were used to detect the selective sweep signals. The intersection of selected parts of the FST and θπ is presented in Figure 6; Supplementary Figure S10. In total, 846 genes were identified in candidate intervals within the SNP corresponding to the gene (Supplementary Table S8). Gene annotation and pathway analysis showed that enrichment of GO terms was significantly concentrated between XBH and XBG cattle, with the three novel genes selected for XBG cattle being enriched in the 18 GO terms with Q < 0.05 (adjusted *p*-value) and concentrated between BS and XBH cattle, with the 23 genes selected for XBH being enriched in the 1 GO term (adjusted *p*-value) (Figure 7; Supplementary Table S9). KEGG analysis showed that nine genes were enriched in five pathways with Q < 0.05 (adjusted *p*-value). Most of the highly enriched pathways included cell adhesion molecules (bta04514), osteoclast differentiation (bta04380), and the insulin secretion pathway (bta04911) (Table 3; Supplementary Table S10).

**TABLE 3:** Pathways significantly enriched between breeds and candidate genes within those pathways.

XBH vs. XBG, XBG selected gene				
Pathway ID	Pathway name	Pathway candidate gene entry ID/KO	*p*-value^a^	Q-value^b^
bta04514	Cell adhesion molecules	bta:525796/KO6797	0.006352	0.006352
KZ vs. XBH, XBH selected gene				
Pathway ID	Pathway name	Pathway candidate gene entry ID/KO	*p*-value^a^	Q-value^b^
bta04380	Osteoclast differentiation	bta:536097/KO6551, bta:529990/KO6551	0.000289	0.018238
bta04911	Insulin secretion	bta:282573/KO4946, bta:505740/KO8045	0.000486	0.018238
		bta:538996/KO5032, bta:532060/KO5004		
XBH vs. KZ, KZ selected gene				
Pathway ID	Pathway name	Pathway candidate gene entry ID/KO	*p*-value^a^	Q-value^b^
bta04340	Hedgehog signaling pathway	bta:522467/KO0312	0.002477	0.036980
bta05217	Basal cell carcinoma	bta:511308/KO0312	0.002958	0.036980

^a^ Statistical test method: hypergeometric test/Fisher’s exact test.

^b^ FDR, correction method: Benjamini and Hochberg.

Abbreviations: KZ, Kazakh; XBG, Xinjiang Brown cattle-grazing type; XBH, Xinjiang Brown cattle-housing type.

### 3.8 Core SNP marker information statistics

Regarding the core loci of the resulting 8379 SNPs, further analysis of SNP distribution in the genome revealed that 88.24% and 1.86% were located in intronic and exonic regions, respectively, and 3.58% and 0.33% were located in intronic and exonic non-coding RNA, respectively; 2.03% and 2.49% were observed in the 5 kb regions upstream and downstream of the transcription start site, respectively, with 0.30% and 1.05% in the 5′ and 3′ UTRs, respectively, and 0.02% in the splice junctions (Supplementary Table S11). Specific information has been provided for these SNP core loci (Supplementary Table S12).

## 4 Discussion

The GBS method is simple, quick, extremely specific, highly reproducible, and may reach important regions of the genome that are inaccessible to sequence capture approaches. GBS libraries based on reducing genome complexity with restriction enzymes (REs), making it feasible for species with high genetic diversity and large genomes (Elshire et al., 2011). SLAF-seq techniques share similarities with GBS in their principles and methods in that they are both part of reduced-representation genome sequencing. SLAF-seq methods, however, are not the same as GBS methods in a few respects. For example, one tag is identified by SLAF-seq roughly every 10 K, the uniform distribution of SLAF tags guarantees that significant chromosomal segments are not overlooked, and SLAF-seq is a cost-effective method since it avoids repeating sequences (Chen et al., 2022b). While both SLAF-seq and WGS can identify SNPs, they greatly differ in terms of cost-effectiveness and missing data. Although SLAF-seq is speedy and inexpensive, its DNA fragmentation stage causes a significant amount of missed data. WGS may be better for small sample sizes since it can yield more information; however, most labs find that a large-scale genotyping project quickly becomes unnecessarily expensive. In contrast, SLAF-seq is an affordable substitute that can yield the same outcomes and detect the same genetic links for a significantly smaller price (Friel et al., 2021). As we gathered a sizable sample size of 130 XBG cattle, the SLAF-seq approach was selected to make the distinction between XBG and XBH cattle clear.

The results show that the four groups of 130 XBG we collected were not clustered in the phylogenetic tree nor the PCA and ADMIXTURE analyses, which is a strong indication that the XBG formed a single population with stable genetic performance after nearly a century of cross breeding. The results also show that the 130 individual cattle are distantly related, indicating that the sample size was both large and representative. These findings represent a strong basis for obtaining reliable genetic analysis results through future analyses.

The ancestors of the Xinjiang Brown cattle, BS and KZ cattle, have been clearly delineated in the global classification of cattle breeds. The world’s cattle breeds are divided into five main categories: European, Eurasian, and East Asian taurines and Chinese and Indian indicines. The BS and KZ breeds are both Eurasian taurines, and their offspring, Xinjiang Brown cattle, also fall within this category (Chen et al., 2018). Our research focused on clarifying the similarities and differences between the two different types of Xinjiang Brown cattle and the genetic relationships of the ancestral species, so no other cattle breeds were included. In utilizing new methods of data merging from the past 2 years, we successfully combined the power of SLAF-seq and WGS methods to exploit a wider range of data sources for research purposes in a cost-effective manner. The results show that XBG and XBH cattle are two different cattle that can be divided into separate groups as per the phylogenetic tree as well as PCA and ADMIXTURE analyses. Furthermore, regarding XBG cattle, the percentage of KZ ancestry was greater than that of BS ancestry; for XBH cattle, the opposite was noted. Software unsuited for unequal sampling (Puechmaille, 2016) and close ancestral populations (Liu et al., 2013; Uren et al., 2020) may have produced less rigorous results, even though the global ancestry proportion was inferred using classical ADMIXTURE. Thus, the local ancestry proportions were estimated by utilizing the fitting software RFMix. One of the two ancestors of the KZ accounts for 10.21% of the inferred ADMIXTURE ancestry. This is most likely because KZ and BS cattle are of the same Eurasian taurine breed or that KZ cattle were inadvertently contaminated with BS genes during the breeding process of the Xinjiang Brown cattle. It was deduced by ADMIXTURE and RFMix that over 95% of the blood in XBH cattle comes from one of the two ancestors and BS. This outcome is in line with the findings of earlier WGS research (Chen et al., 2022a) and indicates that the inferred genetic structure results remained accurate after merging with SLAF-seq data, despite the high loss of SNP loci. However, XBG cattle have a BS genetic lineage of no higher than 40% and a more than 60% KZ genetic ancestry. Having a greater KZ genetic ancestry could explain the better adaptability and grazing abilities of XBG cattle with regards to the local environment compared to XBH cattle. This is consistent with frequent crosses with frozen semen of BS cattle having been performed in the last 20 years, as these crosses were used to improve the productive performance of the minority of XBH. Under housing conditions, XBH cattle are significantly more productive than XBG cattle, but the former’s grazing adaptability is significantly reduced. The majority of XBG cattle are not inseminated using frozen semen from BS because of the grazing conditions and nonetheless maintain a strong grazing performance. Therefore, the XBG breed is more representative of the overall Xinjiang Brown cattle breed in terms of genetic background, genetic diversity, adaptability, and population size. As such, the XBG breed should be protected; otherwise, like the XBH breed, it will eventually lose its uniqueness.

The GO terms identified in this study are mainly related to the biosynthetic and metabolic processes of the molybdenum cofactor. Cattle that consumed fodder high in molybdenum during the 1930s developed a crippling illness. Since molybdate is a common trace element, inducing a dietary molybdenum shortage in plants or animals is difficult. For some animals, particularly sheep and cattle, large molybdenum intakes can produce secondary copper insufficiency, making molybdenum extremely hazardous (López-Alonso and Miranda, 2020). Most of the world’s molybdenum mines are in China, with Xinjiang’s molybdenum resources mainly located in Yining, Bole, and Tacheng (Li et al., 2023). These locations overlap closely with the distribution and grazing pastures of XBG cattle. As such, we hypothesize that XBG cattle may be better adapted to high molybdenum environments than XBH cattle.

When comparing differentially expressed genes between XBH and XBG cattle, cadherin 4 (*CDH4*) was enriched in cell adhesion molecules pathways for the selective sweep in XBG cattle. Cell adhesion molecules are (glyco-) proteins expressed on the cell surface that critically influence a wide array of biological processes that include hemostasis, immune responses, inflammation, embryogenesis, and neuronal tissue development (Montoya et al., 2002; Muller, 2003). The transcriptional level of *CDH4* may serve as an effective diagnostic and prognostic biomarker for renal cell carcinoma patients (Zhou et al., 2020), as it is a novel determinant of osteosarcoma tumorigenesis and metastasis (Tang et al., 2018). This level can downregulate impairments through *in vivo* infiltration and malignancies in patient-derived glioblastoma cells (Ceresa et al., 2019). Moreover, *CDH4* may function as a potential tumor suppressor gene in lung cancer (Li et al., 2017). In conclusion, *CDH4* helps confer disease resistance and may explain the higher level of disease resistance in XBG cattle.

When comparing KZ and XBH cattle, the differentially expressed genes adenylate cyclase 5 (*ADCY5*), ATP binding cassette subfamily C member 8 (*ABCC8*), potassium inwardly rectifying channel subfamily J member 11 (*KCNJ11*), and potassium calcium-activated channel subfamily M alpha 1 (*KCNMA1*) were enriched in insulin secretion pathways for the selective sweep in XBH cattle. Regarding insulin secretion, pancreatic beta cells are specialized endocrine cells that continuously sense the levels of blood sugar and other substrates and, in response, secrete insulin to maintain normal metabolic homeostasis. Glucose-induced insulin secretion and its potentiation constitute the principal mechanism of insulin release (Seino et al., 2010; Rorsman and Braun, 2013). A functional regulatory variant associated with type 2 diabetes is located at the *ADCY5* locus in a pancreatic islet enhancer (Roman et al., 2017). The most frequent genetic cause of hyperinsulinism and neonatal diabetes is pathogenic mutations in *KCNJ11* and *ABCC8*; the subunits of the β-cell ATP-sensitive potassium channel, a crucial element of the glucose-stimulated insulin secretion pathway, are encoded by these genes. Dysregulated insulin secretion results from mutations in these two genes (De Franco et al., 2020; Timmers et al., 2021). Exercise and diet influence insulin sensitivity and secretion (Ding et al., 2019) in XBH cattle with low exercise and high feed energy levels; this may reflect genetic alterations that have occurred to adapt to housing management. In addition, the osteoclast differentiation pathways associated with were also enriched; differential genes included signal regulatory protein β1 (*SIRPB1*), signal-regulatory protein alpha (*SIRPα*), and signal-regulator protein gamma (*SIRPG*). Osteoclasts, multinucleated cells originating from the hematopoietic monocyte-macrophage lineage, are responsible for bone resorption (Nakashima and Takayanagi, 2009; Takayanagi, 2010). A member of the immunoglobulin superfamily, SIRPB1 is a signal regulatory protein that can control receptor tyrosine kinase-coupled signaling. SIRPB1 is a potential oncogene capable of activating Akt signaling to stimulate prostate cancer proliferation (Song et al., 2020), and the *SIRPB1* gene confers susceptibility to Crohn’s disease (Tang et al., 2023). The tumor micro-environment features a marked expression of *SIRPα*, an inhibitory receptor present on myeloid cells, as well as its widely distributed counter-receptor *CD47* (De Vlaminck et al., 2021). Genetically, both *SIRPB1* and *SIRPα* are associated with disease resistance and immunity.

Ultimately, the development of core SNPs for XBG cattle provides a basis for the next step of customizing a solid-phase or liquid-phase gene microarray dedicated to Xinjiang brown cattle for germplasm resource identification, genome-wide association studies research, and genomic selection.

Overall, we comprehensively evaluated the genetic relationship and diversity of XBG cattle compared with two ancestral breeds and another type of the same breed (XBH). Our findings provide new insights into the historical contribution of foreign BS and Chinese KZ breeds to Xinjiang Brown cattle. These findings will help develop a reliable and sustainable strategy for the conservation and improvement of Xinjiang Brown cattle. This study’s results convey that SLAF-seq initially provides very few loci and even fewer loci following data merging, resulting in few enriched GO terms and KEGG pathways. After determining the representativeness and breed significance of XBG cattle, WGS was required to obtain additional loci and information to detail germplasm characteristics.

## Data Availability

The datasets presented in this study can be found in online repositories. The names of the repository/repositories and accession number(s) can be found in the article/Supplementary Material.
