# Corrigendum: Genome-wide survey reveals the genetic background of Xinjiang Brown cattle in China

**DOI:** 10.3389/fgene.2024.1372841

**Published:** 2024-02-28

**Authors:** Xiao Wang, Zhen Ma, Liang Gao, Lixin Yuan, Zhibing Ye, Fanrong Cui, Xiaoping Guo, Wujun Liu, Xiangmin Yan

**Affiliations:** ^1^ College of Animal Science, Xinjiang Agricultural University, Urumqi, China; ^2^ Yili Vocational and Technical College, Yili, China; ^3^ Institute of Animal Science, Xinjiang Academy of Animal Science, Urumqi, China; ^4^ Yili Kazakh Autonomous Prefecture General Animal Husbandry Station, Yili, China

**Keywords:** Xinjiang Brown cattle, specific-locus amplified fragment-sequencing, genetic structure, genetic diversity, candidate genes, ancestry proportion

In the published article, there was an error in the legend for Figure 4 as published. In the phrase “(B) Principal component analysis for the first two PCs of the 178 studied cattle”, 178 needs to be replaced with 177 in order to be consistent with the numbers in the text. The corrected legend appears below.

**FIGURE 4:**
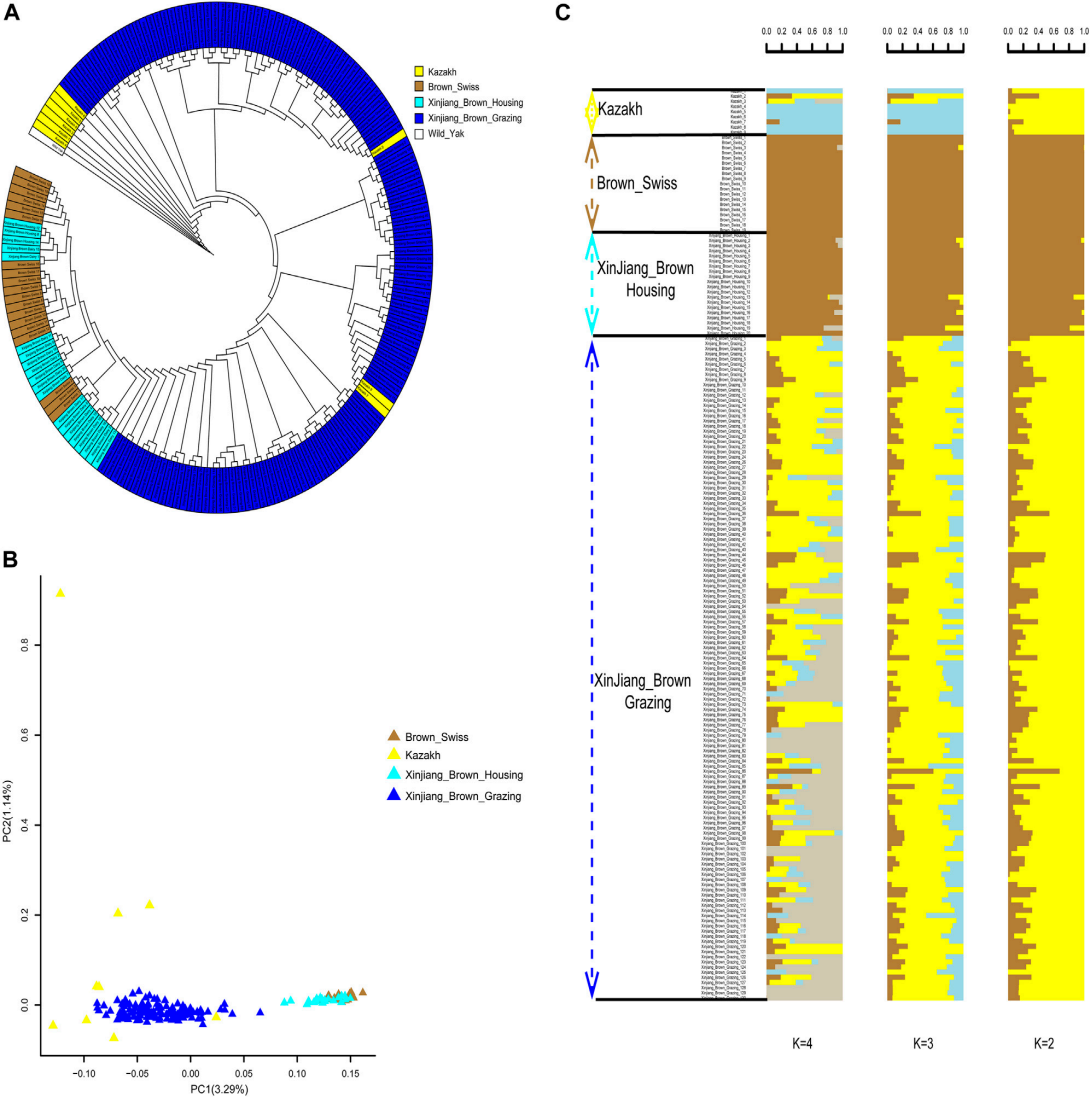
Phylogenetic relationship and population structure of the Xinjiang Brown cattle-grazing type (XBG) cattle and the other three breeds evaluated in this study. **(A)** Neighbor-joining phylogenetic tree constructed from single-nucleotide variant data among four populations. **(B)** Principal component analysis for the first two PCs of the 177 studied cattle. **(C)** ADMIXTURE analysis with four presumed ancestral groups to two presumed ancestral groups (K = from 2 to 4).

“Figure 4. Phylogenetic relationship and population structure of the Xinjiang Brown cattle-grazing type (XBG) cattle and the other three breeds evaluated in this study. **(A)** Neighbor-joining phylogenetic tree constructed from single-nucleotide variant data among four populations. **(B)** Principal component analysis for the first two PCs of the 177 studied cattle. **(C)** ADMIXTURE analysis with four presumed ancestral groups to two presumed ancestral groups (K = from 2 to 4).”

In the published article, there was an error in the legend for Figure 5 as published. In the phrase “Ancestry proportion of the 130 XBG and 20 XBH individuals inferred using RFMix, as based on the reference panels of Kazakh and Brown Swiss cattle”, 130 needs to be replaced with 129, in order to be consistent with the numbers in the text**.** The corrected legend appears below.

**FIGURE 5:**
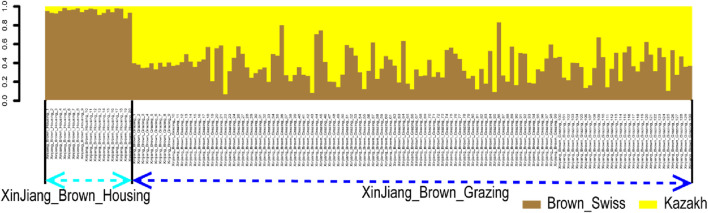
Ancestry proportion of the 129 XBG and 20 XBH individuals inferred using RFMix, as based on the reference panels of Kazakh and Brown Swiss cattle.

“Figure 5. Ancestry proportion of the 129 XBG and 20 XBH individuals inferred using RFMix, as based on the reference panels of Kazakh and Brown Swiss cattle.”

In the published article, there was an error in the **Funding**. “National Agricultural Science and Technology Special Project of China (No. NK2022130302)” is a secret item and its number needs to be deleted. The correct **Funding** statement appears below.

“The author(s) declare financial support was received for the research, authorship, and/or publication of this article. This research was funded by the Xinjiang Uygur Autonomous Region Science and Technology Major Project (No. 2022A02001-1), Xinjiang Brown Cattle Joint Breeding Group Improvement and Enhancement Action Plan Issues (No. 2023XJHN-14), and Xinjiang Agriculture Research System (No. XJARS-XM-08).”

The authors apologize for these errors and state that this does not change the scientific conclusions of the article in any way. The original article has been updated.

