# Supplementary material for: Genome-wide survey reveals the genetic background of Xinjiang Brown cattle in China
Source: Front Genet. 2024 Jan 12;14:1348329. doi: 10.3389/fgene.2023.1348329 (PMC10811208; doi:10.3389/fgene.2023.1348329)
Supplement: Supplementary file 2 [file Image1.pdf]

Supplementary Material

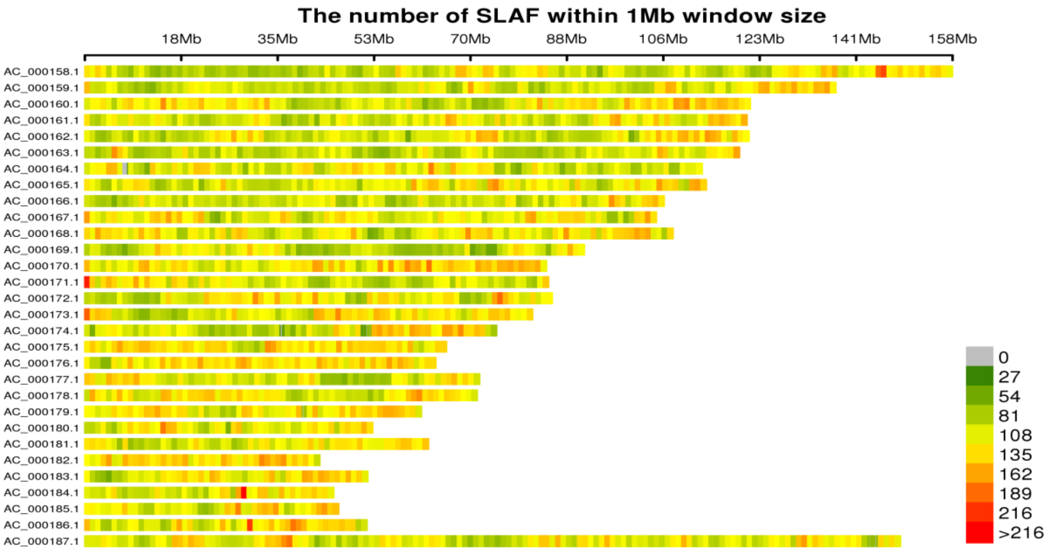

Supplementary Figure S1. Distribution of specific-locus amplified fragment (SLAF) tags on Xinjiang Brown cattle-grazing type chromosomes. Chromosome length is shown on the abscissa: each band represents a chromosome and the genome is divided into 1 Mb segments. Each segment is colored according to the number of SLAF tags: the darker areas are those where SLAF tags are concentrated.

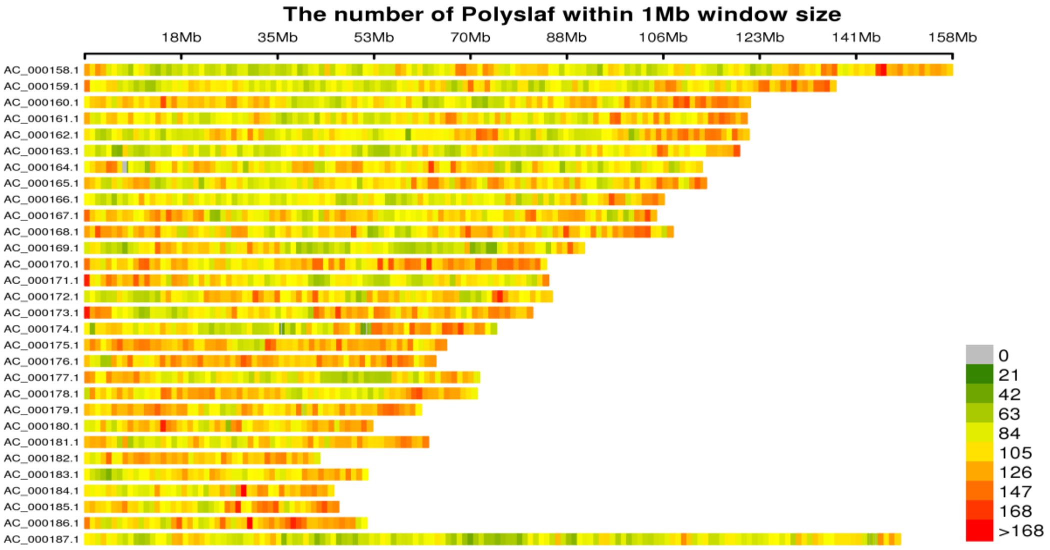

Supplementary Figure S2. Distribution of polymorphic specific-locus amplified fragment (SLAF) tags on Xinjiang Brown cattle-grazing type chromosomes.

Chromosome length is shown on the abscissa: each band represents a chromosome and the genome is divided into 1 Mb segments. Each segment is colored according to the number of SLAF tags: the darker areas are those where SLAF tags are concentrated.

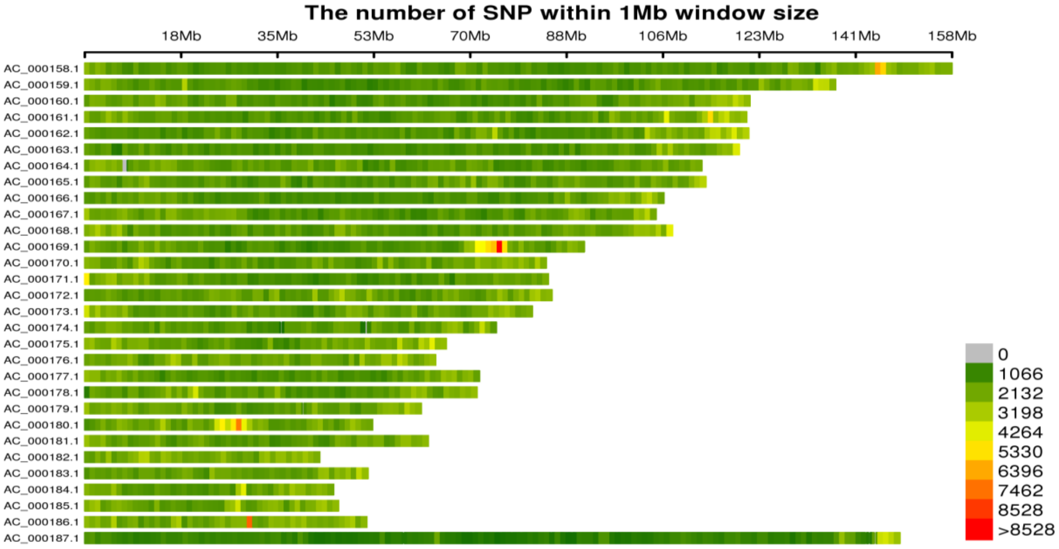

Supplementary Figure S3. Map showing the distribution of single-nucleotide polymorphisms (SNPs) across Xinjiang Brown cattle grazing type chromosomes. Chromosome length is shown on the abscissa: each band represents a chromosome and the genome is divided into 1 Mb segments. Each segment is colored according to the number of SNPs: the darker areas are those where SNPs are concentrated.

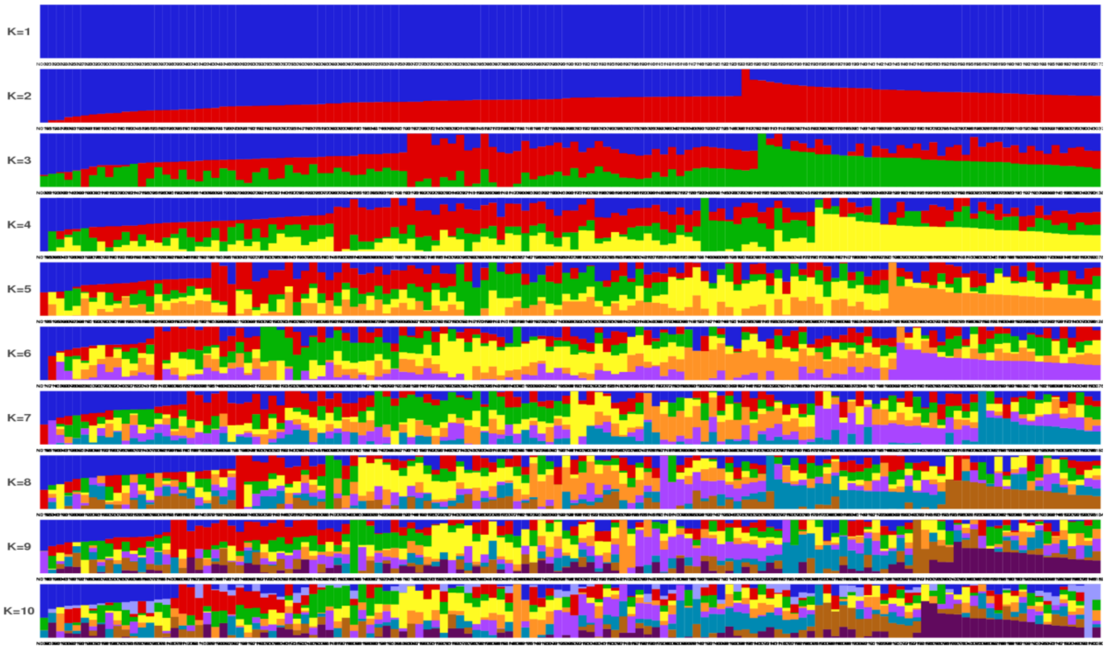

Supplementary Figure S4. ADMIXTURE analysis was performed to estimate the optimum number of clusters (k) in the data set. Ancestry of each sample using k = 1-10 clusters.

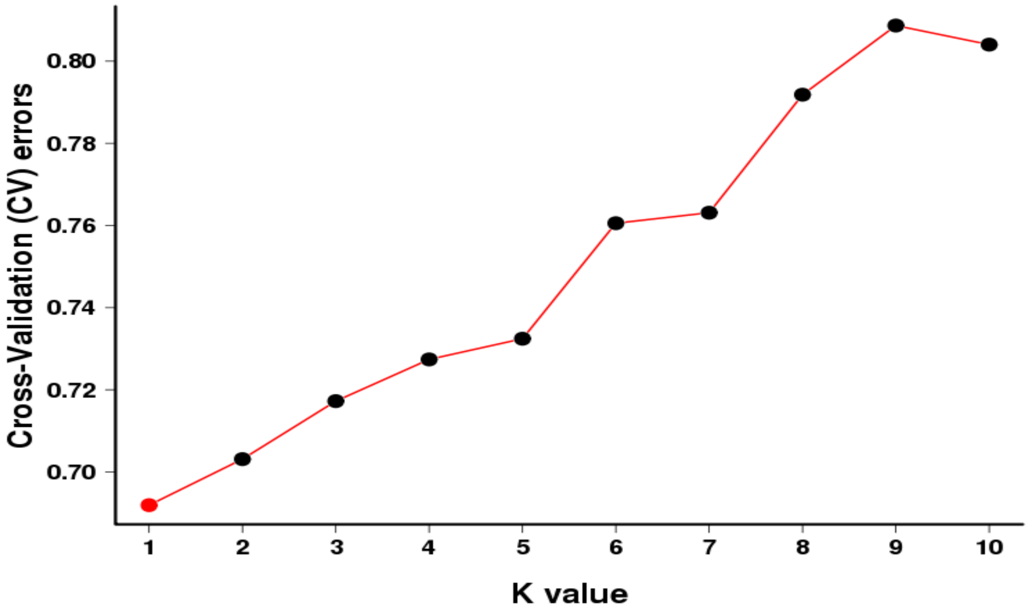

Supplementary Figure S5. Cross validation errors for diverse k values. As shown, k = 1 minimizes the cross-validation error.

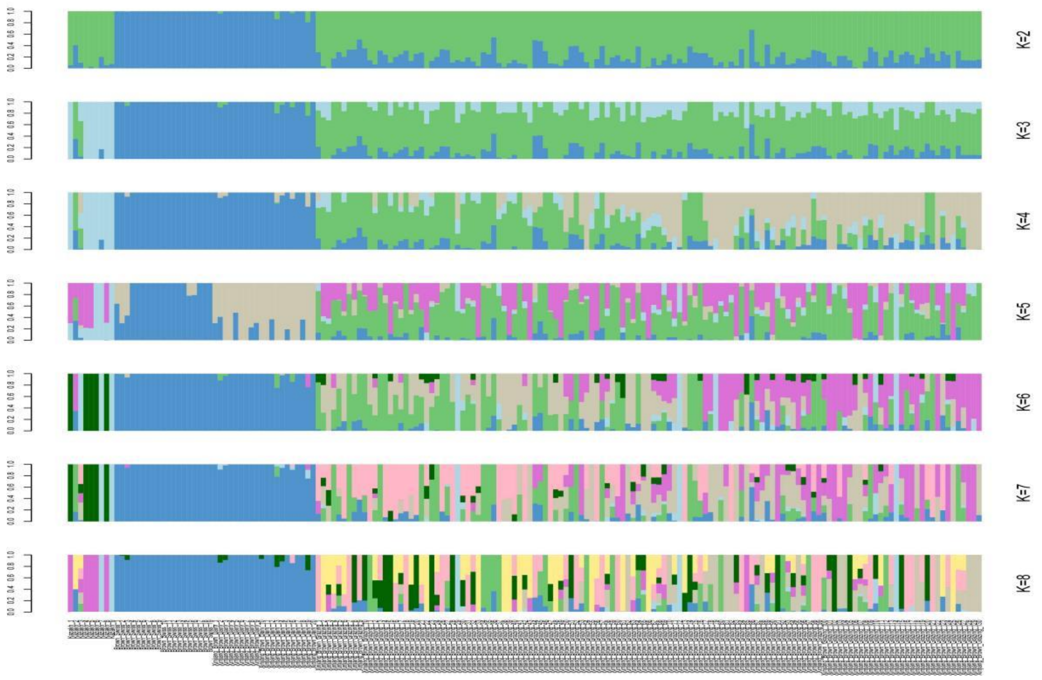

Supplementary Figure S6. An ADMIXTURE analysis was performed to estimate the optimum number of clusters (k) in the data set. Ancestry of each sample using k = 2–8 clusters.

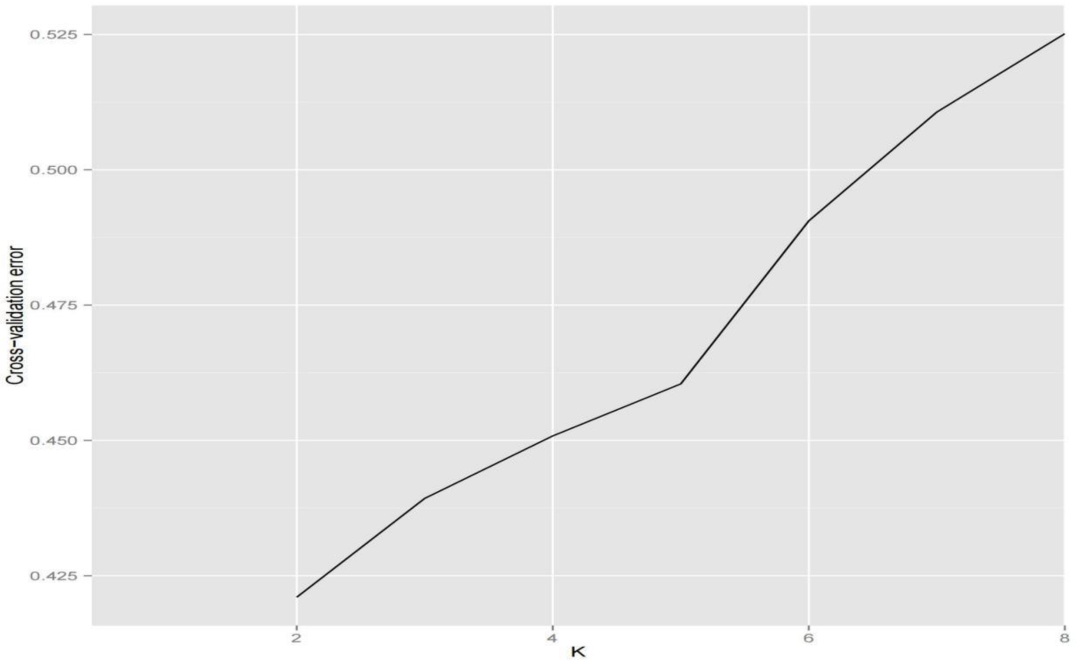

Supplementary Figure S7. Cross validation errors for diverse k values. As shown, k = 2 minimizes the cross-validation error.

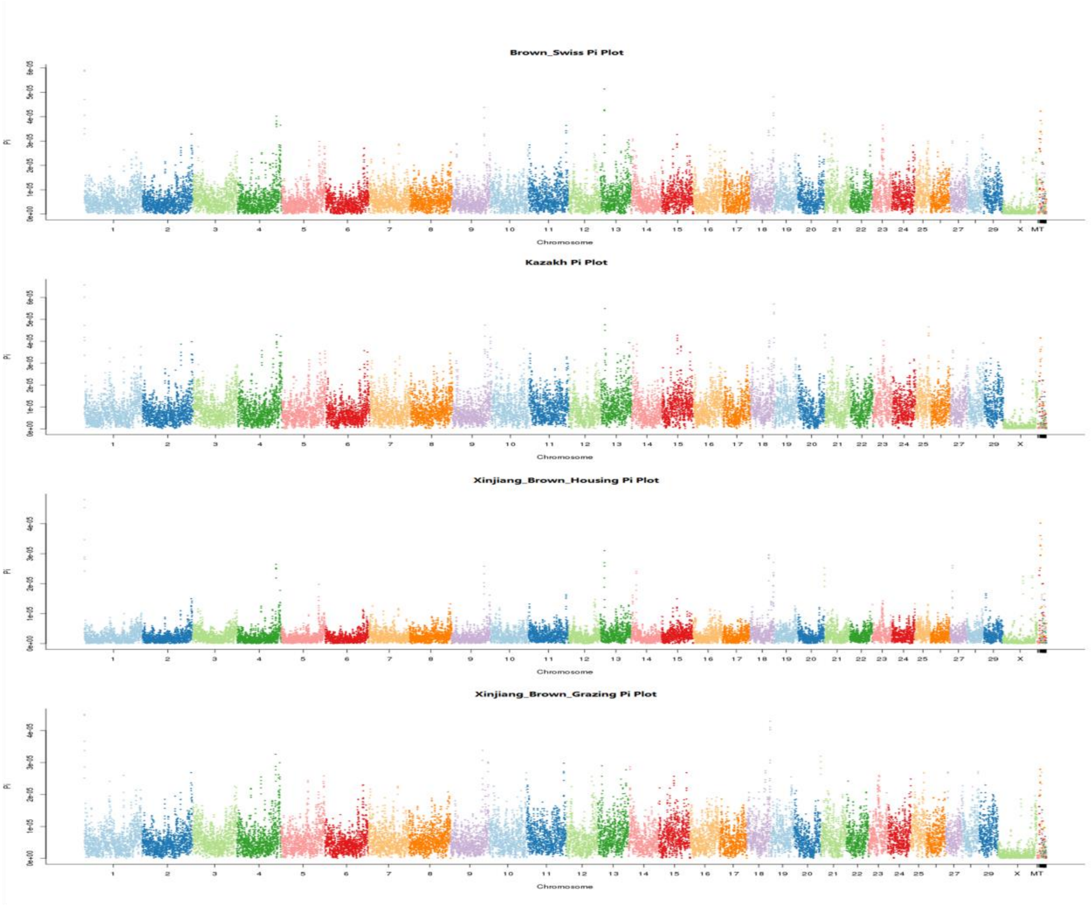

Supplementary Figure S8. Manhattan distribution of the nucleotide diversity (PI) of four breeds of cattle.

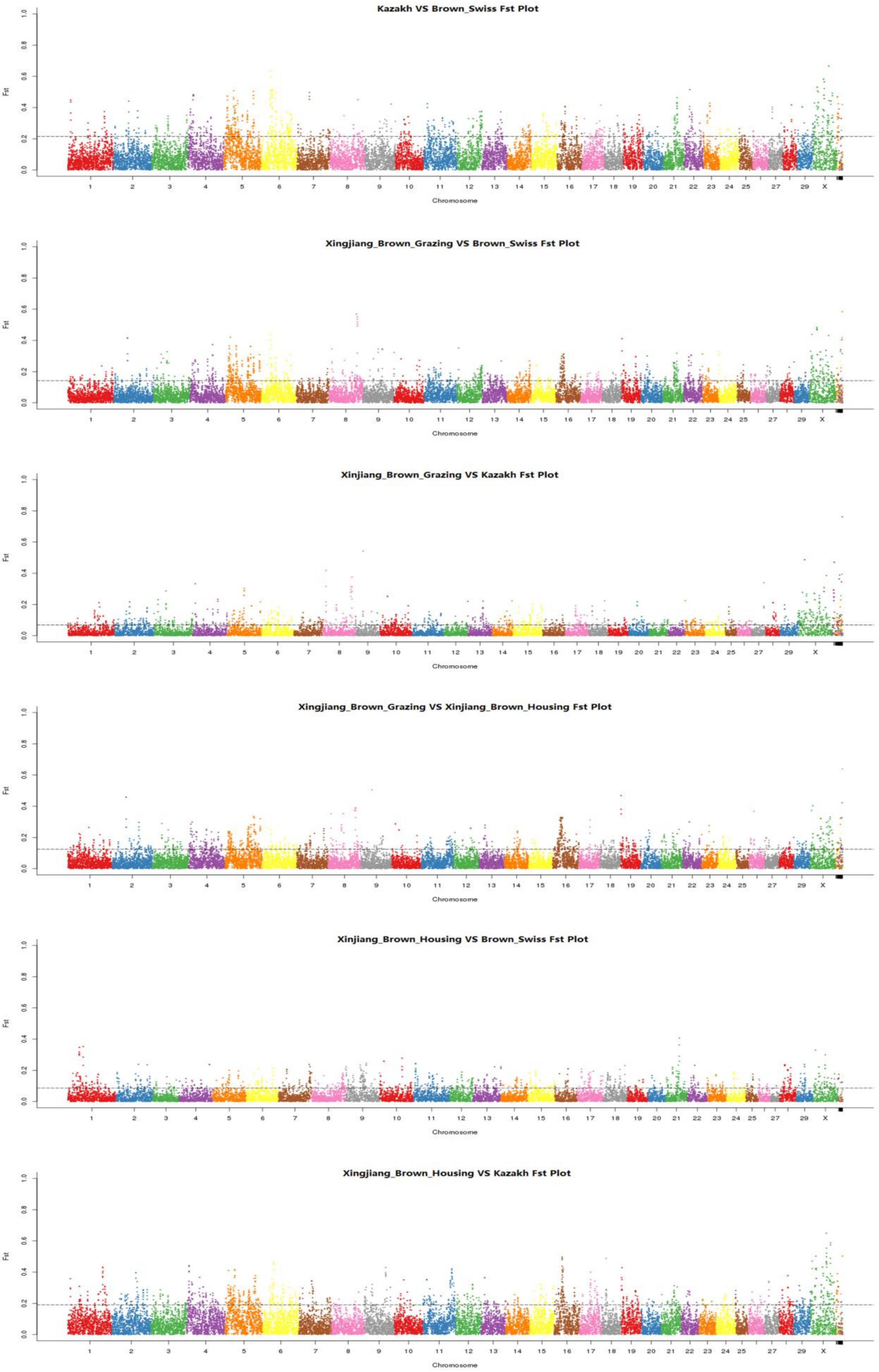

Supplementary Figure S9. Manhattan distribution of the fixation index (FST) between two of the four cattle breeds.

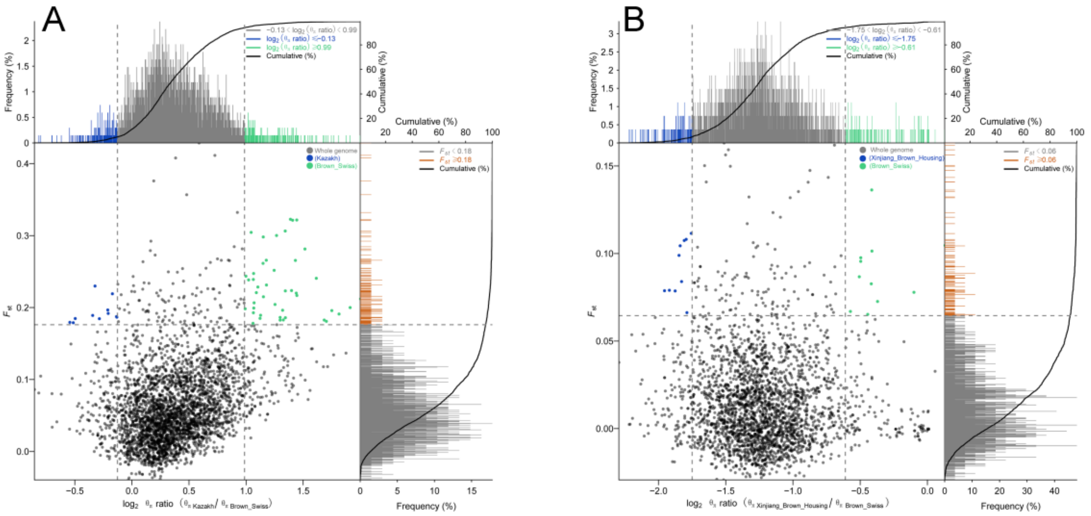

Supplementary Figure S10. Schematic diagrams of selection signal. (A) Kazakh cattle vs. Brown Swiss cattle. (B) Xinjiang Brown cattle-housing type vs. Brown Swiss cattle.
